# When Atrial Fibrillation Meets Alcoholic Liver Cirrhosis: Can Direct Oral Anticoagulants Bridge the Therapeutic Gap?

**DOI:** 10.3390/biomedicines14030531

**Published:** 2026-02-27

**Authors:** Iulia Cristina Marginean, Sergiu Marian Cazacu, Cristina Maria Marginean, Mihaela Popescu, George Alexandru Iacob, Marian Sorin Popescu, Cristin Constantin Vere

**Affiliations:** 1 Doctoral School, University of Medicine and Pharmacy of Craiova, 200349 Craiova, Romania; iulia.cristina18@yahoo.com; 2 Research Center of Gastroenterology and Hepatology, University of Medicine and Pharmacy of Craiova, 200349 Craiova, Romania; vere_cristin@yahoo.com; 3 Department of Internal Medicine, University of Medicine and Pharmacy of Craiova, 200349 Craiova, Romania; popescu.mariansorin@yahoo.com; 4 Department of Endocrinology, University of Medicine and Pharmacy of Craiova, 200349 Craiova, Romania; mihaela.n.popescu99@gmail.com; 5 Department of Radiology and Medical Imaging, Clinical Emergency County Hospital Craiova, 200349 Craiova, Romania; georgeicb5@gmail.com

**Keywords:** ALD, atrial fibrillation, direct oral anticoagulants

## Abstract

A significant clinical challenge is represented by the use of anticoagulants in patients with chronic liver diseases—such as metabolic steatohepatitis (MASH), metabolic associated steatotic liver disease (MASLD), and liver cirrhosis (LC). There is a well-established association between alcohol-related LC and atrial fibrillation (AF). These individuals often require anticoagulation, but treatment must carefully balance the heightened risks of both thrombosis and bleeding. Direct oral anticoagulants (DOACs) are recognized as effective and safe alternatives to warfarin, offering superior stroke prevention and a more favorable safety profile regarding major bleeding. They are generally considered safe for use in patients with LC classified as Child–Pugh A and B—excluding rivaroxaban—but are contraindicated in those with Child–Pugh C cirrhosis. DOACs also offer practical advantages, including convenience of administration, fewer drug interactions, and a high level of safety and efficacy. Comprehensive randomized controlled trials with well-defined cirrhosis stages and standardized anticoagulation protocols are essential to guide clinical decision-making. Until then, a multidisciplinary, individualized approach remains critical in managing patients with both AF and LC. The present review aims to explore the complex interplay between alcohol-related LC and the therapeutic use of direct oral anticoagulants (DOACs), particularly in the presence of cardiovascular risk factors such as atrial fibrillation, and the associated thrombotic complications.

## 1. Introduction

Liver cirrhosis (LC) represents a growing global health burden, with recent studies indicating a rising prevalence [1]. It ranked as the 16th leading cause of disability worldwide and the 7th most disabling condition among adults. Although cirrhosis linked to hepatitis B and C viruses is declining due to improved prevention and treatment, cases associated with alcohol consumption and metabolic associated steatotic liver disease (MASLD) are increasing rapidly. This trend is closely tied to the global increased incidence of metabolic diseases such as obesity and type 2 diabetes, which are key risk factors for MASLD. Consequently, the burden of MASLD-related and alcohol-related LC is expected to continue growing in the coming years [1,2].

Atrial fibrillation (AF) is more common in cirrhotic patients than in the general population, particularly among hospitalized patients. Prevalence estimates vary widely depending on the setting: ICU vs. outpatient, transplant waiting list vs. general cirrhosis patients. Incidence is modest but higher than matched non-cirrhotic cohorts, for instance, in the Korean data, about 3.5 cases per 1000 person-years in cirrhosis, and the severity of liver disease (for example higher MELD score) correlates with higher AF risk [3,4].

Direct oral anticoagulants (DOACs) are recognized as effective and safe alternatives to warfarin (widely used to manage and prevent venous thromboembolism and ischemic stroke in patients with AF), offering superior stroke prevention and a more favorable safety profile regarding major bleeding [4]. The use of DOACs in LC must be highly individualized, balancing the increased thrombotic risk in AF with the elevated bleeding risk due to hepatic dysfunction [4]. Child–Pugh classification is central to guiding therapy, and in advanced disease (Child C), DOACs are contraindicated, with warfarin or no anticoagulation being considered based on bleeding risk and thrombotic history [5,6]. They are generally considered safe for use in patients with LC classified as Child–Pugh A and B—excluding rivaroxaban—but are contraindicated in those with Child–Pugh C cirrhosis. DOACs also offer practical advantages, including ease of administration, fewer drug interactions, and a high level of safety and efficacy. The availability of specific reversal agents, such as idarucizumab for dabigatran, further enhances their safety profile. Moreover, DOACs are associated with improved patient adherence compared to warfarin, as they eliminate the need for routine INR monitoring [5,6].

## 2. Materials and Methods

This narrative review was conducted to synthesize current evidence on the management of AF in patients with LC, with a focus on the role of DOACs. The methodology was designed to comprehensively gather, evaluate, and summarize relevant literature without a formal systematic review protocol, allowing for a broader exploration of the complex pathophysiology and clinical management strategies.

A primary literature search was performed using the PubMed database, screening for papers published before 1 January 2026. The initial search strategy employed a combination of key terms and MeSH headings, including but not limited to “atrial fibrillation,” “liver cirrhosis,” “direct oral anticoagulant,” “DOAC,” “anticoagulation,” “Child-Pugh,” “portal hypertension,” “thrombosis,” “bleeding risk,” “metabolic dysfunction-associated steatotic liver disease,” and “alcohol-associated liver disease.” This initial search was purposefully broad to capture the wide scope of the topic. The reference lists of retrieved articles were subsequently screened to identify additional relevant publications that were not captured in the primary search, ensuring a comprehensive and organic inclusion of foundational and recent studies.

Included literature comprised original research articles (retrospective cohort studies, prospective studies, meta-analyses), clinical guidelines, and authoritative reviews published in English. Priority was given to studies specifically addressing anticoagulation in patients with concomitant AF and LC, as well as foundational papers detailing the pathophysiology of liver disease, hemostatic imbalance, and the pharmacology of anticoagulants. Data pertaining to epidemiology, pathophysiology, clinical trial outcomes, drug metabolism, and management recommendations were extracted, compared, and narratively synthesized to form the basis of this review.

The manuscript was drafted and prepared using Microsoft Word (version 16.59). All graphical figures were created using Affinity Designer (version 2.6.4) and Draw.io (version 29.2.9). Reference management, including the storage, organization, and citation of all the literature, was handled using Zotero (version 6.0.37). The final manuscript underwent proofreading and language refinement assisted by the AI language model DeepSeek (version V3).

## 3. The Global Burden of Alcohol Consumption, a Growing Concern

Alcohol consumption is a major global health concern, accounting for approximately 3.8% of all deaths and 4.5% of global disability [7]. In Europe, the burden is particularly pronounced, with around 6.5% of all deaths attributed to alcohol use [8]. Harmful alcohol consumption—especially in cases of alcohol dependence—is estimated to cause one in seven deaths among men and one in thirteen among women aged 15 to 64 years [9,10]. While mortality from LC has declined in many Western European countries over recent decades, it has risen in several Eastern European countries, as well as in the United Kingdom, Ireland, and Finland [11].

### 3.1. Ongoing Interplays in Alcoholic Liver Disease (ALD)

LC is a complex process involving multiple cell types: both hepatocytes and sinusoidal lining liver cells such as hepatic stellate cells (HSCs), sinusoidal endothelial cells (SECs), and Kupffer cells (KCs). Upon exposure to inflammatory cytokines, these cells—particularly HSCs—become activated and differentiate into myofibroblasts, leading to the deposition of collagen. This fibrotic process disrupts the normal exchange of nutrients between hepatocytes and the sinusoidal blood flow [12,13]. The ongoing interplay between fibrosis, hepatocyte atrophy, and localized regeneration results in the formation of characteristic cirrhotic nodules [14]. Sinusoidal endothelial cells (SECs), are uniquely characterized by fenestrations that facilitate the exchange of fluids and nutrients between the blood and hepatocytes [14]. Chronic alcohol consumption can lead to the loss of these fenestrations, which contributes to perisinusoidal fibrosis and disrupts normal liver microcirculation [15].

Alcohol-related liver damage is driven by several mechanisms, including the release of pro-inflammatory cytokines (like tumor necrosis factor-alpha [TNF-α], interleukin-6 [IL-6], and interleukin-8 [IL-8]), oxidative stress, lipid peroxidation, and the toxic effects of acetaldehyde. These factors collectively lead to hepatocellular inflammation, apoptosis, and progressive fibrosis [16,17].

A hallmark of alcoholic liver disease (ALD), steatosis, is often accompanied by hepatocellular necrosis and inflammation. Cytokines such as TNF-α, IL-6, and IL-8 are considered to be central mediators of liver injury, contributing to hepatocyte apoptosis, cytotoxic hepatomegaly, and progressive hepatotoxicity [18,19].

### 3.2. Excessive Lipid Accumulation in Hepatocytes

Excessive lipid accumulation in hepatocytes occurs when the liver increases the uptake of circulating fatty acids (FAs) or activates de novo lipogenesis, leading to the synthesis of lipids within the liver itself [20,21]. Acetaldehyde plays an essential role in this process. especially by promoting lipolysis in adipose tissue, thereby increasing the influx of free fatty acids (FFAs) to the liver [22]. These FFAs are taken up by hepatocytes through fatty acid transporters, including fatty acid transport proteins (FATPs) and fatty acid translocase (FAT/CD36). Notably, elevated expression of FATP2, FATP5, and FAT/CD36 has been described in liver tissues of ALD mouse models [22].

Acetaldehyde also interferes with the 5′-adenosine monophosphate-activated protein kinase (AMPK) pathway, which normally regulates lipid metabolism by suppressing lipogenic transcription factors [23,24]. Disruption of this pathway leads to the upregulation of sterol regulatory element-binding protein 1c (SREBP-1c) that further activates the expression of key lipogenic enzymes such as acetyl-CoA carboxylase 1 (ACC1), fatty acid synthase (FASN), and stearoyl-CoA desaturase 1 (SCD1) [23,24].

LIPIN1 (a gene encoding a key enzyme in lipid metabolism) also plays a crucial role in hepatic lipid homeostasis, acting as a transcriptional coactivator, as well as functioning as a magnesium-dependent phosphatide phosphatase, promoting the synthesis of triglycerides and phospholipids [25]. Ethanol exposure has been shown to increase LIPIN1 expression via SREBP-1c activation in an AMPK-dependent manner, further contributing to lipid accumulation in hepatocytes [25].

Hepatic lipid disposal primarily involves two key processes: mitochondrial β-oxidation and the export of excess triglycerides via very-low-density lipoproteins (VLDLs) [26]. The RNA splicing factor SFRS10 (serine/arginine-rich splicing factor 10) promotes the generation of the LIPIN1α isoform through exon skipping [26]. However, studies by Yin et al. [27] have shown that ethanol increases microRNA-217 levels, which suppress SIRT1-mediated activation of SFRS10. This results in a shift toward the LIPIN1β isoform, increasing the LIPIN1β/α ratio. As a result, LIPIN1’s function favors lipid biosynthesis over mitochondrial fatty acid (FA) β-oxidation [27].

Peroxisome proliferator-activated receptor alpha (PPARα) is a key transcription factor that regulates genes involved in mitochondrial FA oxidation [28]. Acetaldehyde impairs PPARα function by reducing its DNA-binding capacity [29], leading to downregulation of target genes like carnitine palmitoyltransferase 1 (CPT1), a critical enzyme responsible for transporting fatty acids into mitochondria for β-oxidation [30]. In addition, autophagy plays an important role in clearing ethanol-induced lipid droplet accumulation. However, chronic ethanol exposure impairs autophagy, likely due to acetaldehyde-mediated suppression of AMPK activity [31,32].

Since hepatocellular fat accumulation is the first hallmark of ALD, a deeper understanding of lipid metabolism in hepatocytes is crucial, providing valuable opportunities for early therapeutic interventions in individuals at risk of disease progression [33].

### 3.3. Dysregulated Immune System

Emerging research shows that chronic alcohol consumption disrupts the integrity of the intestinal barrier, particularly by impairing tight and adherens junctions within the colonic mucosa. This compromise allows the translocation of lipopolysaccharide (LPS), a bacterial endotoxin, into the systemic circulation [34,35]. Acetaldehyde, a key ethanol metabolite, exacerbates this effect by upregulating microRNA-212 in enterocytes and downregulating zona occludens-1 (ZO-1), a critical component of tight junctions [36]. LPS and acetaldehyde activate Kupffer cells—the liver’s resident macrophages—triggering the release of reactive oxygen species (ROS) and chemokines [36]. These mediators promote the recruitment and infiltration of immune cells, including monocytes and bone marrow-derived neutrophils, into the liver [34]. ROS produced by Kupffer cells further activate the toll-like receptor 4 (TLR4)/mitogen-activated protein kinase (MAPK)/nuclear factor-kappa B (NF-κB) signaling pathway, amplifying the inflammatory response [37]. In parallel, free fatty acids (FFAs) have also been shown to activate the TLR4/NF-κB pathway in vitro, leading to increased inflammatory mediators such as cyclooxygenase-2 (COX-2) in macrophages [38]. In support of the central role of TLR4 in alcohol-induced liver injury, in vivo studies have demonstrated that TLR4-deficient mice are resistant to ethanol-induced hepatic steatosis [39].

Elevated cytokines—tumor necrosis factor-alpha (TNFα), interleukin-1 beta (IL-1β), and interleukin-8 (IL-8)—are commonly observed in patients with ALD [40]. Exposure to oxidative ethanol metabolites activates the NF-κB signaling pathway, leading to increasing TNFα production in macrophages [17,41], activation of NF-κB in Kupffer cells and, finally, systemic release of pro-inflammatory mediators [42]. Additionally, partial activation of NF-κB signaling can result from the inhibition of SIRT1, a natural antagonist of NF-κB [43]. This pathway mediates reactive oxygen species (ROS)-triggered inflammatory responses via downstream signaling effectors, including intercellular adhesion molecule 1 (ICAM1). ICAM1 facilitates interactions between hepatocytes and neutrophils, thereby promoting neutrophil-driven hepatocyte damage [44].

Patients with alcoholic hepatitis exhibit elevated circulating antibodies targeting hydroxyethyl radical (HER)-protein adducts and lipid peroxidation-derived aldehydes such as malondialdehyde (MDA) [45]. Elevated levels of anti-HER and anti-MDA antibodies are related to activation of peripheral CD4+ T cells. Additionally, oxidative metabolism of ethanol impairs proteasome function in macrophages, leading to defective antigen presentation [46,47]. This disruption compromises the activity of both macrophages and dendritic cells [46]. Furthermore, chronic alcohol consumption reduces the population of F4/80+ macrophages expressing major histocompatibility complex class I (MHC-I) and class II (MHC-II) molecules [48].

Fatty acids increase the sensitivity of hepatocytes to inflammatory signals while simultaneously impairing their response to protective factors like signal transducer and activator of transcription 3 (STAT3) [49,50]. Additionally, ROS produced during ethanol metabolism rapidly increase hepatocyte membrane permeability, leading to excessive accumulation of cytoplasmic iron [51] and intensified lipid peroxidation. This cascade ultimately results in widespread hepatocyte death [51].

#### Induction of Fibrosis

Extensive hepatocyte loss initiates the liver’s fibrotic repair response. In alcoholic steatohepatitis, hepatic stellate cells (HSCs) become activated and transform into the most relevant producers of extracellular matrix components, such as collagens and fibronectin, driving the progression of liver fibrosis [52]. Protein adducts formed by acetaldehyde and lipid peroxidation-derived aldehydes, including malondialdehyde (MDA), stimulate pro-fibrogenic signaling pathways within these activated HSCs [40]. In vitro studies demonstrate that acetaldehyde released by hepatocytes can enter HSCs, inducing the expression of genes encoding type I collagen [53]. Acetaldehyde regulates collagen gene expression via a protein kinase C (PKC)-dependent mechanism. In human HSCs, PKC activates extracellular signal-regulated kinase (ERK) and phosphoinositide 3-kinase (PI3K), which leads to the phosphorylation of p70 S6 kinase (p70S6K) and ultimately promotes collagen gene upregulation [54].

Another fibrosis-promoting mechanism driven by acetaldehyde involves the transforming growth factor-beta (TGFβ) pathway, a key player in liver fibrosis development [55]. In human hepatic stellate cells (HSCs), early exposure to acetaldehyde increases the transcription of genes encoding type I collagen and fibronectin independently of TGFβ [56]. However, during the later stages of treatment, TGFβ-dependent processes are activated, including the secretion of latent TGFβ1 and upregulation of the type II TGFβ receptor [57].

Moreover, lipopolysaccharides (LPSs) from the gut amplify HSC activation by enhancing their responsiveness to both acetaldehyde and TGFβ [58,59].

Increasing evidence indicates that acetaldehyde promotes the activation of hepatic stellate cells (HSCs) in ALD by inducing oxidative stress [60]. Reactive oxygen species (ROS) generated during ethanol metabolism via CYP2E1 increase collagen synthesis in HSCs co-cultured with hepatocytes [61,62]. Similarly, acetaldehyde suppresses the transcriptional activity of peroxisome proliferator-activated receptor gamma (PPARγ) in activated hepatic stellate cells [63]. Additionally, acetaldehyde forms adducts with glutathione (GSH), reducing its antioxidant capacity [64]. Nuclear erythroid 2-related factor 2 (NRF2), a transcription factor activated by oxidative stress, enhances the expression of antioxidant genes [65]. Increased NRF2 expression has been shown to alleviate ALD by reducing oxidative stress [66], while NRF2 deficiency worsens ALD progression [66].

Chronic alcohol consumption impairs the anti-fibrotic function of natural killer (NK) cells, which normally target activated hepatic stellate cells (HSCs), thereby accelerating liver fibrosis. This suppression occurs through interactions involving the TNF-associated apoptosis-inducing ligand (TRAIL) and its receptor, along with interferon gamma (IFNγ) signaling [67]. Additionally, interleukin-22 (IL-22), produced by both NK cells and T helper cells, inhibits acetaldehyde-induced activation and proliferation of HSCs [68]. IL-22 promotes the nuclear translocation of NRF2 in HSCs, leading to their deactivation by arresting the cell cycle at the G1/S phase. In mouse models of carbon tetrachloride (CCl_4_)-induced liver fibrosis, IL-22 overexpression induces HSC senescence through upregulation of p53, a key regulator of cellular senescence [69]. Beyond its anti-fibrotic role, IL-22 also exerts anti-apoptotic, antioxidant, and pro-regenerative effects against alcohol-induced liver injury, supporting its potential as a therapeutic agent in ongoing clinical trials for ALD [70].

**Figure 1:**
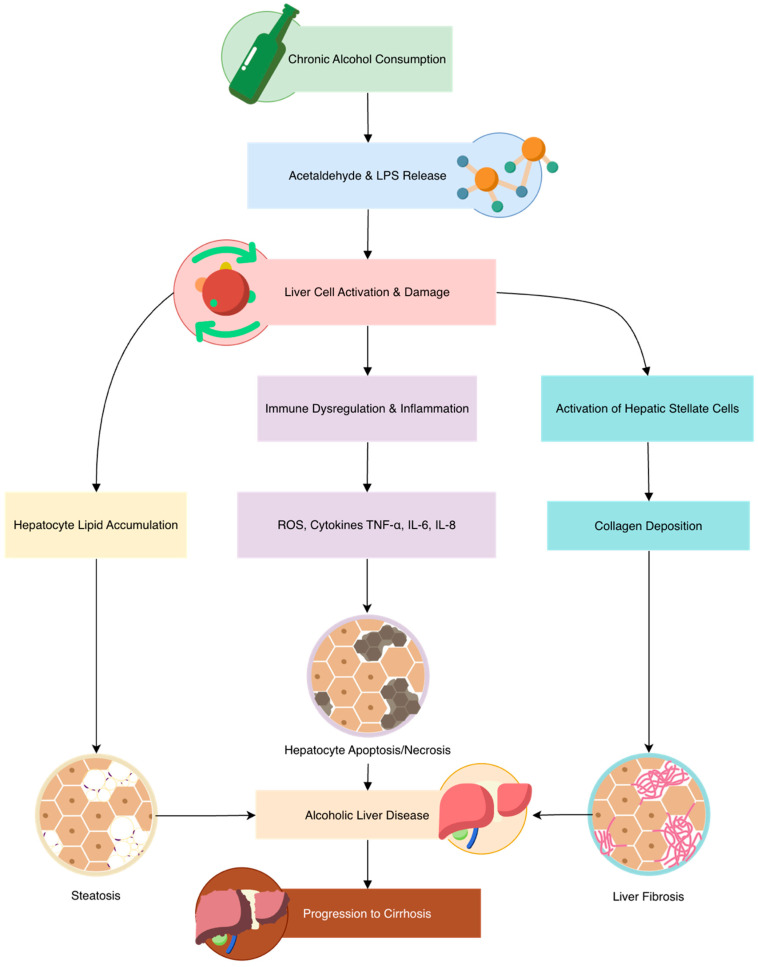
Pathophysiology of alcohol-related liver damage and fibrosis.

ROS act as key activators of intracellular fibrogenic mechanisms in HSCs, such as ERK, protein kinase B (PKB/Akt), and tissue inhibitor of metalloproteinase 1 (TIMP1) [71]. The fibrogenic role of ROS is supported by findings that ROS-scavenging enzymes can suppress hepatic fibrosis in ALD animal model studies [72]. These results highlight the potential of targeting oxidative stress, for example with antioxidants, as a promising strategy to alleviate fibrosis associated with ALD (Figure 1).

While the molecular mechanisms outlined above provide important insights into alcohol-related liver injury, their clinical relevance to anticoagulation lies in the net effect on hemostatic balance. Progressive fibrosis disrupts hepatic synthetic function, reducing production of both procoagulant and anticoagulant factors; endothelial dysfunction and portal hypertension create localized hemodynamic alterations affecting bleeding risk; and the severity of liver dysfunction, rather than specific molecular pathways, remains the primary determinant of anticoagulant safety and selection. Thus, this pathophysiological understanding underscores why Child–Pugh classification, platelet count, and variceal screening are central to clinical decision-making in this population.

## 4. Atrial Fibrillation, a Pathology of Alarmingly Increasing Incidence

AF can lead to serious complications such as blood clot formation, stroke, heart failure, and other cardiovascular events. It is projected that by 2030, over 12 million people will be affected by AF [73]. Studies show that approximately 15% to 20% of stroke patients also have this arrhythmia. Due to the significant risk of clot formation in AF, anticoagulant therapy is essential for affected patients. If left untreated, AF substantially increases the risk of death from cardiovascular causes—doubling the likelihood—and is associated with a fivefold higher risk of stroke [74]. Amidst the global trends of aging populations and increased life expectancy despite chronic diseases, the prevalence of AF is rising significantly, affirming its status as a worldwide epidemic [75]. Data from the Framingham Heart Study reveal that the prevalence of AF has tripled over the past fifty years [76]. The Global Burden of Disease study estimated that approximately 46.3 million people worldwide currently live with AF [76]. Projections of lifetime risk also reflect this upward trend: in 2004, the lifetime risk of developing AF was estimated at about 1 in 4 for white men and women over 40 years old [77]. A decade later, these estimates have shifted to roughly 1 in 3 for white people and 1 in 5 for black individuals [77].

In the United States, an estimated 3 to 6 million individuals currently live with atrial AF, with projections suggesting this number could rise to between 6 and 16 million by 2050 [75,78]. In Europe, the prevalence of AF among those over 55 years old was around 9 million in 2010, and it is expected to rise to 14 million by 2060 [79]. Similarly, estimates indicate that by 2050, at least 72 million people across Asia will be diagnosed with AF, with approximately 3 million experiencing AF-related strokes [80].

The prevalence of AF is significantly lower in Asian and African individuals compared to those of European descent, despite a higher burden of comorbidities among African-descended populations [81]. This disparity is likely influenced by a combination of genetic, socioeconomic, and environmental factors, although these influences have not yet been fully explored [82]. The Multi-Ethnic Study of Atherosclerosis (MESA) found that Hispanics, Asians, and African Americans over 65 years old experience 46% to 65% fewer AF episodes than non-Hispanic whites [83,84]. Similarly, an analysis of over 600,000 patients within the Veterans Affairs healthcare system revealed that the age-adjusted prevalence of AF in Caucasians was nearly double that of other ethnic groups [85].

While lower AF rates in these populations have been partly attributed to underdiagnosis due to limited healthcare access, genetic studies have identified specific single-nucleotide polymorphisms that partially explain the increased susceptibility to AF seen in Americans of European ancestry compared to African Americans [86]. Additionally, analyses from the Cardiovascular Health Study (CHS) and the Atherosclerosis Risk in Communities (ARIC) study, which utilized ancestry-informative markers, linked higher European ancestry with an elevated risk of AF [87]. However, systematic data on electrophysiological differences across ethnicities remain limited, underscoring a significant knowledge gap in this area [88,89].

Over the past decade, awareness and detection of AF have improved markedly, which is critical since roughly one third of AF patients are asymptomatic [81]. As a result, the global burden of AF is likely underestimated. Additionally, the widespread availability of portable rhythm monitoring devices, driven by consumer adoption, is expected to further increase AF diagnosis rates and raise awareness worldwide [81].

### 4.1. Underlying Conditions Related to Atrial Fibrillation

The majority of persistent and permanent AF cases are linked to underlying conditions such as hypertension, valvular heart disease, ischemic heart disease, and other structural cardiac abnormalities. However, approximately 15% of AF cases are classified as “lone AF,” occurring in the absence of any obvious underlying heart disease. Early research identified a genetic locus on chromosome 10 (10q22-q24) associated with familial AF exhibiting an autosomal dominant inheritance pattern [90]. Nonetheless, familial AF shows considerable heterogeneity in its presentation [91]. More recent studies have uncovered the genetic complexity underlying familial AF. For example, a mutation in the gene encoding the α subunit of the cardiac IKs channel on chromosome 11 has been reported in a family with persistent AF [92]. This mutation enhances the channel’s function, leading to shortened atrial refractoriness—i.e., the heart’s reduced ability to regain electrical stability between beats—thus predisposing affected individuals to persistent AF [92,93].

The underlying mechanisms of AF in individuals with seemingly healthy hearts remain less well understood. While some overlap exists, pulmonary vein triggers tend to play a more significant role in younger patients with structurally normal hearts who experience brief episodes of paroxysmal AF. In contrast, for patients with structural heart disease and persistent or permanent AF, the presence of an abnormal atrial substrate is likely the dominant factor driving the arrhythmia [94].

AF risk increases significantly after the age of 65, a concern that is amplified by demographic projections estimating the population over 65 will rise from 12% in 2010 to 22% by 2040 [95]. Chronic subclinical inflammation—a persistent, low-grade activation of the immune system that accompanies biological aging across multiple organs—is a shared feature of both AF and advanced age. Inflammation is closely associated with endothelial dysfunction, collagen degradation, increased activity of TGF-β1, and remodeling of the extracellular matrix, all playing critical roles in AF pathogenesis [96].

### 4.2. Pathophysiology of Atrial Fibrillation

AF and LC share a deep, cross-organ connection rooted in common underlying disease processes [97]. While cirrhosis affects the liver and AF affects the heart, both are driven by chronic inflammation, fibrosis, and oxidative stress. Indeed, cirrhosis is now recognized as an independent risk factor for new-onset AF, mediated through cirrhotic cardiomyopathy [98].

#### 4.2.1. Chronic Systemic Inflammation

Both conditions exist in a state of persistent, low-grade systemic inflammation [99]. In LC, the damaged liver releases inflammatory mediators (TNF-α, IL-1, IL-6) into the circulation, while gut-derived endotoxins from bacterial translocation amplify this response [100]. In AF, systemic inflammation acts directly on the atrial myocardium, inducing fibrosis, electrical remodeling, and autonomic imbalance characterized by increased sympathetic tone. Elevated C-reactive protein and cytokine levels serve as biomarkers for both advancing liver fibrosis and AF development [98].

#### 4.2.2. Fibrosis and Structural Remodeling

Fibrosis represents the pathological hallmark of both diseases—the replacement of functional tissue with collagenous scar [101]. In LC, portal hypertension and inflammation activate hepatic stellate cells, driving excessive extracellular matrix deposition [102]. In AF, identical molecular pathways (notably TGF-β) activate cardiac fibroblasts within the atria, producing interstitial fibrosis that disrupts electrical conduction [101]. Profibrotic signals that scar the liver—including Galectin-3 and TGF-β—simultaneously promote atrial remodeling, creating an arrhythmogenic substrate [103].

#### 4.2.3. Renin–Angiotensin–Aldosterone System Activation

Both conditions feature RAAS overactivation. In LC, splanchnic vasodilation reduces effective arterial blood volume, triggering RAAS activation that promotes fluid retention and accelerates hepatic fibrogenesis. In AF, RAAS activation—particularly angiotensin II—drives cardiac hypertrophy and fibrosis. Accordingly, ACE inhibitors and ARBs have shown promise in both slowing cirrhosis progression and reducing AF recurrence [104].

#### 4.2.4. Oxidative Stress

Imbalance between reactive oxygen species and antioxidants is central to both conditions. Impaired liver function reduces antioxidant capacity while endotoxemia increases ROS production, perpetuating cellular damage and fibrosis [105]. Concurrently, elevated ROS levels damage cardiac myocytes, disrupt calcium handling, and accelerate fibrotic remodeling [106]. Mitochondrial dysfunction mediates much of this oxidative injury in both organs.

#### 4.2.5. Hemodynamic Changes

LC characteristically produces a hyperdynamic circulation—high cardiac output with low systemic vascular resistance [107]. In AF, the heart operates under persistently stressed, hyperdynamic conditions, promoting atrial enlargement. This mechanical stretch directly contributes to rhythm disorders by altering atrial tissue architecture [108].

#### 4.2.6. Shared Risk Factors

While LC and AF affect different organ systems, they frequently intersect through common metabolic and lifestyle risk factors. Chief among these is chronic alcohol abuse, which is directly toxic to both hepatic parenchyma and cardiac conduction tissue [109]. Beyond alcohol, the global epidemic of metabolic syndrome creates a powerful common pathway: obesity, hypertension, type 2 diabetes, and dyslipidemia independently drive both MASLD progression to cirrhosis and the atrial stretch, inflammation, and electrical remodeling that precipitate AF [110]. Moreover, combined metabolic and alcohol-related insults (MetALD) synergistically promote atrial remodeling and systemic inflammation, producing an AF risk that exceeds that of MASLD alone [111].

These shared mechanisms explain why LC and AF frequently coexist and why management strategies must address both organs simultaneously—a theme central to the anticoagulation decisions discussed in this review.

### 4.3. Alcohol Consumption and Atrial Fibrillation

Alcohol consumption is widespread in Western countries, with nearly half of the American population drinking regularly. The American Heart Association recommends limiting alcohol intake to a maximum of two drinks per day for men and one drink per day for women, preferably consumed with meals [112]. A comprehensive meta-analysis found that moderate alcohol consumption—defined as one drink per day—does not significantly increase the risk of developing AF [112]. However, in the U.S., about 17% of adult drinkers (approximately 37 million people) engage in binge drinking [113]. A recent meta-analysis revealed that each additional daily alcoholic drink raises the risk of AF by nearly 8%, demonstrating a clear linear and dose-dependent relationship [114]. Evidence from the ARIC study further highlights that both the amount and duration of alcohol consumption increase susceptibility to AF [115]; conversely, abstinence is linked to a reduced risk of AF [116].

Prolonged ethanol exposure induces notable changes in cardiac electrophysiology, including lengthened His bundle–ventricle (HV) intervals, widened QRS complexes, and disrupted atrial myocyte action potentials. These alterations increase vulnerability to arrhythmias in both animal and human studies [117]. Equally important, heavy alcohol intake exerts direct toxic, inflammatory, and oxidative damage on the left atrial myocardium. Findings from the Framingham Heart Study (FHS) link alcohol consumption to enlargement of the left atrium and a higher risk of developing AF [118]. Additionally, alcohol affects the left ventricle by promoting remodeling and increasing left ventricular pressure, thereby contributing to diastolic dysfunction [119].

Recent research highlights that a significant reduction in alcohol intake or abstinence effectively reduces AF recurrence in habitual drinkers [116]. This benefit likely results not only from halting alcohol’s direct proarrhythmic effects but also from weight loss, as alcohol is calorie-dense (7 kcal/g). Excessive alcohol consumption can lead to weight gain and hypertension, both of which are recognized triggers for AF onset [120].

### 4.4. Imbalance Between Thrombosis and Bleeding in Liver Disease

The liver is the primary site for the synthesis of multiple coagulation factors, although factor VIII is also produced extrahepatically. All pro- and antifibrinolytic proteins are synthesized in the liver, both by hepatocytes and endothelial cells, which makes fibrinolysis particularly vulnerable to hepatic dysfunction [121]. Patients with cirrhosis frequently exhibit profound disturbances in their hemostatic system, with abnormalities affecting all phases—primary hemostasis, secondary hemostasis, and fibrinolysis. These alterations contribute to a paradoxical increased risk of both bleeding and thrombosis [121].

The increased risk of thrombosis in patients with liver disease is largely due to reduced levels of natural anticoagulants alongside elevated circulating procoagulants. Impaired liver function leads to decreased synthesis of key anticoagulants such as protein C and antithrombin, which significantly contributes to a heightened thrombotic tendency [122]. Additionally, patients with liver disease often experience increased platelet aggregation driven by elevated activity of von Willebrand factor (vWF) and reduced levels of ADAMTS13—a protease that regulates vWF function [123,124]. This imbalance enhances platelet binding to glycoprotein Ib and collagen, increasing the effectiveness of high-molecular-weight vWF multimers in supporting clot formation. Proteases like plasmin and elastase can also degrade vWF in the setting of liver disease, further influencing its multimeric structure, which is normally regulated by ADAMTS13-mediated proteolysis [125].

Since the liver produces almost all coagulation factors (except factor VIII and vWF), their plasma levels decline in liver disease, increasing bleeding risk. Reduced levels of fibrinogen and factors II, V, VII lead to prolonged prothrombin time (PT), while decreased activity of factors II, V, IX, X, XI, and XII results in an extended activated partial thromboplastin time (aPTT) [126,127].

Increased fibrinolysis has also been observed in liver disease, primarily due to elevated levels of tissue plasminogen activator and reduced concentrations of plasmin inhibitor and thrombin-activatable fibrinolysis inhibitor (TAFI) [128]. In patients with compensated cirrhosis, the balance between procoagulant and anticoagulant forces is delicate. However, this fragile equilibrium can be easily disrupted by precipitating factors such as hepatic decompensation, sepsis, fluid shifts, renal impairment, or invasive procedures—potentially tipping the balance toward either thrombosis or bleeding complications [129].

The three major anticoagulant proteins—named protein C, protein S, and antithrombin—are significantly reduced in LC, related to both decreased hepatic synthesis and increased consumption [130]. When a strong thrombotic stimulus is present, thrombin generation can still occur despite the diminished levels of procoagulant factors [131].

Furthermore, in LC, both the number and function of platelets are decreased. However, the elevated levels of von Willebrand factor (vWF) can partially compensate for the defects in primary hemostasis caused by thrombocytopenia and platelet dysfunction, by enhancing platelet adhesion [132]. Some mechanisms have been proposed to explain the increasing vWF levels in liver disease, including endothelial dysfunction, stimulated hepatic synthesis of vWF, and delayed clearance of the factor [133].

Endothelial function plays a crucial role in maintaining hemostatic balance. Therefore, localized endothelial dysfunction can lead to a hypercoagulable state in a specific anatomical region, even in the presence of a systemic prothrombotic environment [134]. Both intrahepatic and extrahepatic endothelial dysfunction contribute to the development of portal hypertension. Within the intrahepatic microcirculation, hypoactive endothelial cells increase vascular resistance, primarily through reduced nitric oxide (NO) production—an early trigger of portal hypertension (PHT) [135,136]. Once established, PHT impacts not only the hepatic vasculature but also the systemic and splanchnic circulations. It promotes arterial vasodilation and the formation of collateral vessels, resulting in increased blood flow through the portal vein, which in turn further exacerbates portal hypertension [136,137].

In contrast to the hypoactive endothelial cells within the liver, endothelial cells in the splanchnic and systemic circulations become hyperactive, leading to excessive NO production [138]. Additionally, sinusoidal endothelial cell dysfunction facilitates hepatic inflammation and plays a key role in modulating liver immune tolerance, acting as a primary mediator of hepatic immune homeostasis [136].

**Figure 2:**
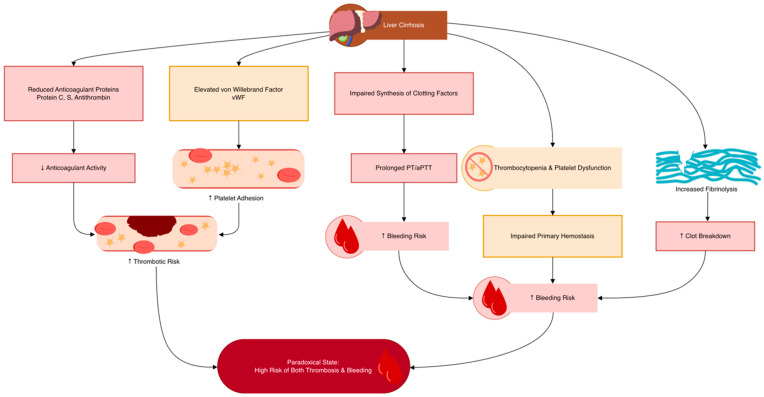
Hemostatic imbalance in liver cirrhosis.

As the liver progressively loses its ability to synthesize coagulation-related proteins, the delicate balance between bleeding and thrombosis becomes increasingly unstable. This paradoxical state predisposes patients with liver failure to both hemorrhagic and thrombotic complications [139,140] (Figure 2).

## 5. When Atrial Fibrillation Meets Liver Cirrhosis

A higher prevalence of AF has been observed in individuals with LC, regardless of its underlying etiology [109]. In a retrospective study of 1727 patients with liver disease proposed for liver transplantation, newly diagnosed AF was found in 11.2% of those with cirrhosis (*p* < 0.001) [141]. The risk of AF also increased in parallel with the severity of liver disease, as evaluated by the Model for End-Stage Liver Disease (MELD) score [142]. Similarly, data from a large national patient cohort showed that individuals with LC have a significantly higher risk of developing AF compared to controls [109]. Moreover, AF has been identified as a predictor of both morbidity and mortality in LC patients [143].

The management of AF in patients with cirrhosis must be dynamically stratified according to disease stage and stability, as the risk–benefit balance for both rate control and anticoagulation shifts along the clinical spectrum [98]. In patients with compensated, stable cirrhosis—where hepatic synthetic function is preserved and portal hypertension is minimal—thromboembolic risk often outweighs bleeding risk, allowing for cautious anticoagulation [144]. Adjusted-dose DOACs may be appropriate in this population, given their favorable safety profiles in Child–Pugh A and select B patients [145]. In decompensated or unstable cirrhosis—characterized by ascites, variceal bleeding, or jaundice—the clinical picture becomes a tightrope walk. Hemodynamic instability and systemic inflammation can trigger AF, while concurrent coagulopathy (elevated INR) and thrombocytopenia create a precarious equilibrium where patients face simultaneous risks of thrombosis and life-threatening hemorrhage [125]. During these unstable phases, anticoagulation is typically contraindicated [146]. Management shifts to addressing underlying triggers (e.g., infection, electrolyte imbalance) and implementing rate control strategies. Anticoagulation may be reconsidered only if the patient returns to a compensated, stable state following multidisciplinary evaluation of portal hypertension and bleeding risk [146]. This clinical stratification aligns with Child–Pugh classification: compensated cirrhosis corresponds largely to Child–Pugh A, while decompensated disease encompasses Child–Pugh B and C, with the latter representing the most unstable end of the spectrum.

The development of AF in cirrhosis patients is closely linked to abnormal autonomic nervous system function. This autonomic dysfunction, a result of LC and portal hypertension, is associated with elevated levels of neuropeptides, such as vasoactive intestinal peptides (VIP), or cytokines including IL-6, IL-8, and TNF-α, as well as oxidative stress markers and fibrosis-related factors like Galectin-3 [142,147]. Liver disease itself is recognized as a notable predictor for the development of new-onset atrial fibrillation, particularly in association with conditions such as nonalcoholic fatty liver disease (NAFLD), nonalcoholic steatohepatitis (NASH), and alcohol-associated liver disease (ALD) [148,149]. Notably, NAFLD and NASH are expected to contribute significantly to cardiovascular complications, including AF [150]. Furthermore, excessive alcohol consumption is a well-established risk factor for AF, primarily through its role in alcoholic cardiomyopathy, increased sympathetic nervous system activity, and subsequent left atrial enlargement [151].

### 5.1. Managing Anticoagulation in Cirrhotic Patients

Vitamin K antagonists (VKAs) have traditionally been the cornerstone of antithrombotic therapy for AF, with the international normalized ratio (INR) serving as the standard measure to monitor their therapeutic effect. More recently, direct-acting oral anticoagulants (DOACs) are preferred for non-valvular AF. However, patients with LC were systematically excluded from the key clinical trials evaluating these agents, primarily due to concerns about impaired hemostasis in these individuals [3,98].

Managing anticoagulation in patients with both AF and LC poses a considerable and noteworthy clinical challenge. LC involves a complex and dynamic imbalance between procoagulant and anticoagulant factors, resulting in a fragile hemostatic equilibrium where both bleeding and thrombotic risks are elevated [125]. This delicate balance is further complicated by thrombocytopenia caused by splenic sequestration, reduced thrombopoietin production, platelet dysfunction, altered drug interaction, impaired synthesis of protein-bound medications, and the presence of gastroesophageal varices [110].

Although routine hemostasis tests, ref. [125] such as prolonged INR, typically indicate a tendency toward impaired clotting, individuals with liver disease paradoxically face a considerable risk of thrombotic events as well [127,152,153]. A large retrospective study using administrative data revealed a significant finding: chronic liver conditions, including viral hepatitis, hepatoma or LC, not only raise the risk of bleeding but also predict a higher likelihood of ischemic cerebrovascular events, strokes, and related complications. As a result, individuals with both AF and liver disease are at a heightened risk of ischemic cerebrovascular events [154,155].

Several studies have identified a link between MASLD and an increased risk of stroke, with stroke risk rising progressively alongside higher fatty liver index values. Therefore, patients with MASLD should receive thorough counseling and close monitoring to evaluate and reduce their stroke risk [155]. Additionally, independent research has shown that MASLD is strongly associated with a greater likelihood of developing atrial fibrillation, a relationship that is especially pronounced in individuals with normal or lower body weights [156,157].

While the CHA_2_DS_2_-VASc and HAS-BLED scores are foundational tools for guiding anticoagulation decisions in the general population, their utility becomes significantly constrained in patients with cirrhosis, as they fail to capture the unique and dynamic hemostatic profile of liver disease [158].

The CHA_2_DS_2_-VASc score may underestimate true thromboembolic risk in this cohort because it does not account for the procoagulant imbalance often present in advanced cirrhosis—where reduced production of natural anticoagulants (protein C and S) can paradoxically increase thrombotic potential despite a prolonged INR [159].

Conversely, the HAS-BLED score is inherently flawed in cirrhotic patients. It penalizes patients for an elevated “labile INR,” which in this context reflects baseline synthetic dysfunction rather than anticoagulation control and does not accurately predict bleeding risk [160]. Moreover, HAS-BLED overlooks the most significant source of hemorrhage in these patients—portal hypertension and its sequelae, such as esophageal varices [161]—which require specific endoscopic risk stratification that falls entirely outside the score’s parameters [158,162].

Consequently, over-reliance on these conventional cardiac scores without integrating hepatology-specific factors can lead to either undertreatment of atrial fibrillation due to a perceived prohibitive bleeding risk or inappropriate initiation of anticoagulation in a patient with untreated high-risk varices [163].

### 5.2. Indications for Oral Anticoagulation Therapy

Managing anticoagulation in patients with chronic liver conditions poses significant clinical challenges due to their elevated risk of bleeding [164]. This heightened risk stems from impaired hepatic synthetic function, the presence of varices, and thrombocytopenia, all of which are common in progressive liver disease. At the same time, these patients also face an increased risk of thromboembolic events, particularly ischemic stroke [146].

Recent studies indicate that in patients with atrial fibrillation and cirrhosis, the use of direct oral anticoagulants (DOACs) does not significantly reduce bleeding complications compared to vitamin K antagonists (VKAs). Importantly, DOACs are not recommended for individuals with Child–Pugh Class C cirrhosis. Rivaroxaban is contraindicated in patients classified as Child–Pugh B or C [145,165]. However, in patients with milder hepatic damage (Child–Pugh Class A and B), the pharmacokinetic profiles of apixaban and rivaroxaban are generally comparable to those seen in individuals without liver dysfunction [145].

Among the different direct oral anticoagulants (DOACs), hepatic excretion varies significantly: approximately 20% for dabigatran, 65% for rivaroxaban, 50% for edoxaban, and 75% for apixaban. In contrast, warfarin undergoes complete hepatic metabolism, with 100% liver-based clearance (see Table 1). These variations suggest that DOACs, compared to warfarin, may offer more predictable pharmacokinetics in patients with LC [3].

**Figure 3:**
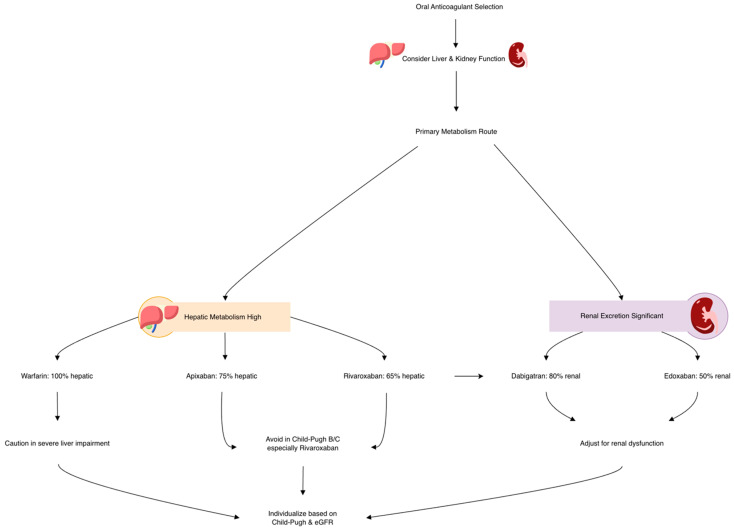
DOAC vs. warfarin: metabolism and selection.

The comparative metabolism and selection criteria for DOACs versus warfarin are summarized in Figure 3. A comprehensive retrospective study of a U.S. national database that examined patients predominantly with class A (Child–Pugh) cirrhosis who developed AF found that the use of DOACs was associated with a diminished all-cause mortality risk, compared to no anticoagulation [144]. Another retrospective cohort study involving 9056 patients with LC and comorbid AF, all with a CHA_2_DS_2_-VASc score ≥2, evaluated the effectiveness of antithrombotic strategies. Patients were grouped based on treatment: antiplatelet therapy, warfarin, and patients without any anticoagulation [98]. The subgroup of patients with both AF and LC who received no antithrombotic therapy had a considerable higher susceptibility of stroke compared to patients without cirrhosis. Notably, those treated with warfarin showed a statistically significant reduction in ischemic stroke rates, whereas stroke rates in the antiplatelet and no-anticoagulation groups were comparable, indicating limited benefit from antiplatelet therapy in this population [98,166].

The comparative efficacy and safety of DOACs versus warfarin in patients with cirrhosis and AF have been evaluated in numerous meta-analyses of observational data, as summarized in Table 2. However, readers should note that these meta-analyses draw from overlapping primary cohorts—predominantly large Asian administrative databases—which may limit the independence of these analyses and their generalizability to Western populations.

### 5.3. Oral Anticoagulant Drugs in Liver Disease and Reversal Agents

Before initiating OAC in patients with known or suspected liver disease, it is essential to perform a comprehensive workup including liver enzyme tests, platelet count, serum creatinine, and also coagulation profiles. These parameters should be continuously monitored during therapy. In cases of thrombocytopenia (e.g., platelet counts between 50,000 and 70,000/mm^3^), the initiation of anticoagulation may need to be delayed, depending on the patient’s thrombotic risk [145].

Patients at risk should also undergo evaluation for esophageal varices or other high-risk bleeding lesions before starting OACs, and screening for alcohol misuse should be part of standard care, with cessation support offered as needed [178]. Prior to initiating oral anticoagulant therapy, all patients with liver damage should be assessed for alcohol use and offered appropriate cessation support. It is essential to educate patients on both the potential risks and benefits of anticoagulation therapy and to actively involve them in shared decision-making on its initiation and choice of agent [175].

**Figure 4:**
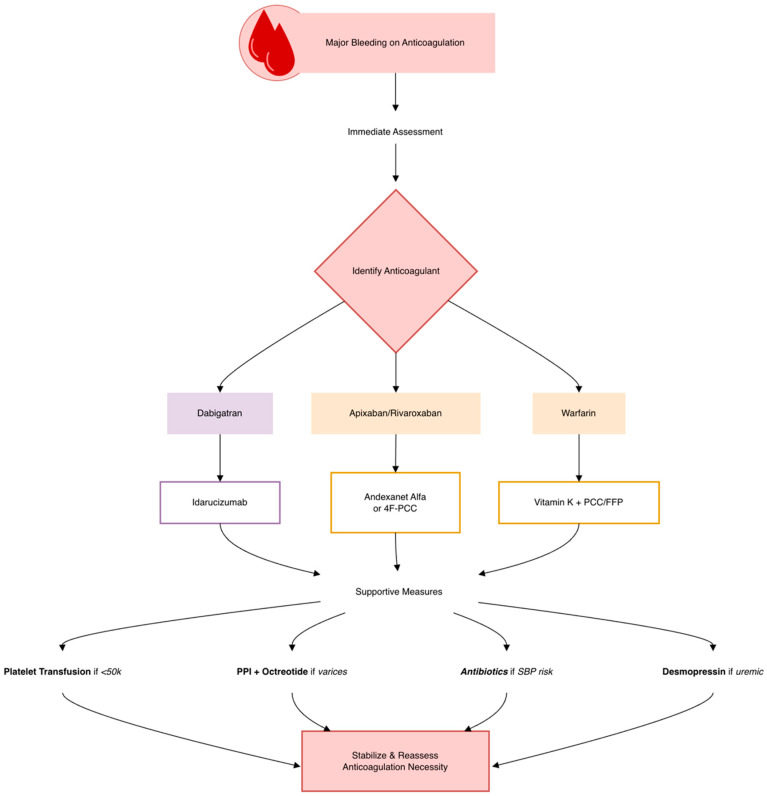
Clinical management and reversal strategies for bleeding.

Patients with recent major bleeding, persistent coagulopathy, or known high-risk hemorrhagic lesions (such as large varices) require individualized anticoagulant strategies [169] (Figure 4). Historically, warfarin has been the default OAC in patients with liver impairment. However, in selected patients with slight hepatic dysfunction (Child–Pugh A), DOACs may be considered without dose adjustment. In more severe hepatic impairment (Child–Pugh C), warfarin is generally preferred, though in cases where warfarin is not viable, cautious use of apixaban, dabigatran, or edoxaban in patients with Child–Pugh B may be considered—with close monitoring and multidisciplinary oversight. Early collaboration between cardiology and hepatology/gastroenterology is key to optimizing OAC use in this complex patient population [179].

If a patient with liver disease experiences major bleeding within two hours of anticoagulant ingestion, administration of activated charcoal is recommended [146,180].

Idarucizumab (Praxbind) is a fully humanized monoclonal antibody fragment specifically designed to reverse the anticoagulant effects of dabigatran, having been approved by both the FDA based on the results of a phase III clinical trial [6].

Reversal agents for other direct oral anticoagulants (DOACs) are also under investigation. These include andexanet alfa, a recombinant modified factor Xa decoy that neutralizes factor Xa inhibitors such as rivaroxaban and apixaban, and ciraparantag, a small synthetic molecule capable of reversing the effects of both factor IIa and Xa inhibitors [181].

In cases of severe thrombocytopenia accompanied by ongoing bleeding, platelet transfusion should be considered. Proton pump inhibitors can be co-administered with somatostatin analogs, such as octreotide, to help reduce portal venous pressure and control bleeding [181].

Additionally, prophylactic antibiotic therapy is recommended to prevent spontaneous bacterial peritonitis (SBP), as studies have demonstrated that short-term antibiotic use in cirrhotic patients with gastrointestinal bleeding not only reduces the incidence of bacterial infections but also improves survival outcomes [169].

Desmopressin, an endothelial stimulant that increases levels of factor VIII and von Willebrand factor, may be used to increase platelet function, particularly in patients with liver disease associated with hepatorenal syndrome [146].

Decisions about anticoagulation must be individualized. An esophagogastroduodenoscopy is recommended at the time of cirrhosis diagnosis to detect high-risk bleeding lesions associated with portal hypertension [182], and in liver transplantation recipients, anticoagulation may be considered to maintain or restore patency of the portal vein, as an unobstructed main portal vein is associated with improved post-transplant survival [183].

In the broader population of patients with both AF and venous thromboembolism (VTE), DOACs have proven effective in reducing the risk of stroke and thrombotic events with an acceptable safety profile [184]. Nonetheless, patients with impaired liver function have largely been omitted from clinical trials evaluating DOACs for stroke and VTE prevention. This omission is significant, as all currently approved DOACs rely to some extent on hepatic metabolism. In the setting of liver dysfunction, this can lead to increased plasma drug concentrations and decreased production of coagulation factors, along with a heightened risk of bleeding [184].

Additionally, several DOACs interact with cytochrome P450 enzymes for their metabolism—enzymes whose activity is often impaired in liver disease [185,186]. Apixaban and rivaroxaban are primarily metabolized via the cytochrome P450 system, making them more susceptible to altered pharmacokinetics in this population. In contrast, dabigatran and edoxaban are less reliant on cytochrome P450 enzymes for metabolism. Liver disease can also reduce biliary excretion of DOACs, and in cases complicated by hepatorenal syndrome or chronic kidney disease, this can be problematic. Furthermore, liver dysfunction affects albumin synthesis. Since some DOACs are highly protein-bound, alterations in albumin levels may increase the proportion of unbound (active) drug, further amplifying bleeding risk [187].

As a result, establishing the most appropriate anticoagulation strategy for both atrial fibrillation and venous thromboembolism patients, in the context of impaired liver function, remains complex and insufficiently defined [145].

Recent studies explore the use of direct oral anticoagulants (DOACs) and conventional antithrombotic drugs—including vitamin K antagonists and heparins—in patients with LC and atrial fibrillation [98]. A large retrospective study conducted on more than 2400 patients with LC and AF, comparing DOAC versus warfarin, concluded that all major digestive bleeding events were significantly lower in the DOAC group [188].

A recent systematic review and meta-analysis of six cohort studies, including 41,954 patients with AF and liver disease treated with DOACs and warfarin, found that anticoagulation therapy was correlated with reduced mortality, with DOACs having a significantly lower risks of major bleeding and gastrointestinal bleeding compared to warfarin [189].

Another recent study conducted by Lee et al. in 2022 concluded that DOACs seem to be associated with greater efficacy and increased safety outcomes in patients with AF and LC, compared to warfarin [171]. Huang ZC et al. showed in a meta-analysis of six studies conducted on 41,859 patients a beneficial effect of DOACs vs. warfarin in patients with liver disease and AF [169].

Cohort studies, including systematic reviews and meta-analyses, indicate that anticoagulation in patients with both LC and AF is associated with a reduced risk of stroke, without a significantly increased risk of bleeding compared to patients without anticoagulation therapy [169,189].

**Figure 5:**
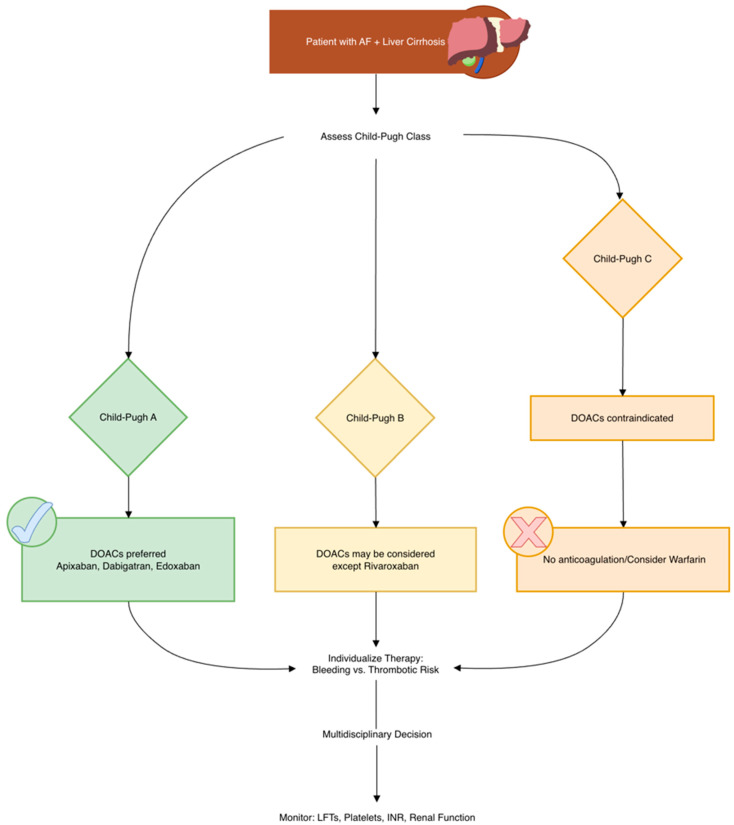
Anticoagulation decision pathway in AF and cirrhosis.

Therefore, DOACs have demonstrated a protective effect against ischemic stroke and systemic embolism in cirrhotic patients with non-valvular atrial fibrillation [180] (Figure 5).

## 6. Discussions

The management of AF in patients with LC represents a significant clinical dilemma, characterized by the need to balance an elevated thrombotic risk against a concomitantly heightened bleeding tendency. This review has synthesized current evidence on the pathophysiological interplay between these conditions and the evolving role of direct oral anticoagulants (DOACs) in bridging this therapeutic gap.

The association between LC and AF is multifactorial and bidirectional. Cirrhosis, particularly of alcoholic or metabolic origin, promotes a pro-inflammatory and pro-fibrotic state that contributes to cardiac structural remodeling and autonomic dysfunction, thereby increasing AF susceptibility. Conversely, AF exacerbates the hemodynamic instability in cirrhotic patients with portal hypertension, potentially worsening outcomes [143]. This complex relationship underscores that the co-existence of AF and LC is not merely coincidental but pathophysiologically interlinked, necessitating integrated management strategies.

A central challenge in anticoagulating these patients lies in the “rebalanced hemostasis” of cirrhosis, a precarious equilibrium where deficits in procoagulant factors coexist with deficiencies in natural anticoagulants, creating a substrate for both hemorrhage and thrombosis [125,127]. Traditional markers like INR are poor predictors of bleeding risk in this population and do not reflect the true thromboembolic potential [125,153]. This paradox renders clinical decision-making particularly complex.

The accumulated evidence from observational studies and meta-analyses, as summarized in this review, suggests that DOACs (apixaban, dabigatran, edoxaban) present a viable and often preferable alternative to vitamin K antagonists (VKAs) in patients with compensated cirrhosis (Child–Pugh A and B) [167,170,172]. The data consistently indicate that DOACs are associated with a reduction in major bleeding events, particularly intracranial hemorrhage, and may offer superior efficacy in preventing ischemic stroke, without increasing all-cause mortality [167,170,174]. The concern for gastrointestinal bleeding—a significant threat in patients with portal hypertension—is underscored by real-world studies showing that upper GI bleeding in patients on antithrombotic therapy carries a substantial mortality risk, a finding that was notably persistent even during the COVID-19 pandemic [190]. This highlights the critical importance of selecting anticoagulants with favorable GI safety profiles and implementing proactive management strategies for varices in this vulnerable cohort. The safety of DOACs in procedural contexts is further supported by real-world data from the Italian IRIS registry, which prospectively followed 250 patients undergoing catheter ablation on rivaroxaban and reported no major bleeding events during 12-month follow-up, with rare thromboembolic complications [191]. While this population differs from cirrhotic patients, these findings add to the broader real-world evidence supporting DOAC safety in procedural settings, a relevant consideration given that cirrhotic patients may require interventions such as variceal banding or paracentesis while anticoagulated.

Their fixed dosing and lack of need for routine monitoring offer practical advantages, potentially improving adherence in a population burdened by multiple comorbidities [5,6].

However, this favorable profile is not uniform across all DOACs or all stages of liver disease. The pharmacokinetic reliance on hepatic metabolism varies, with rivaroxaban (65%) and apixaban (75%) being more dependent than dabigatran (20%) or edoxaban (50%) [3]. Consequently, rivaroxaban is contraindicated in Child–Pugh B and C cirrhosis, while the others require caution and individualized dosing in Child–Pugh B, and are generally contraindicated in Child–Pugh C [145,165]. In decompensated cirrhosis, the altered drug metabolism, hypoalbuminemia, and frequent renal impairment significantly increase the risk of drug accumulation and bleeding, making VKAs with careful INR monitoring or even forgoing anticoagulation the more prudent options in many cases [146,179].

The decision to anticoagulate must therefore be highly personalized, guided by a thorough assessment of both thrombotic risk (using scores like CHA_2_DS_2_-VASc) and liver disease-specific bleeding risk. Essential pre-therapy workup includes evaluation for esophageal varices, assessment of platelet count, and measurement of renal function [146,178]. A multidisciplinary approach involving hepatologists and cardiologists is crucial for optimizing outcomes [160]. For patients on DOACs who experience major bleeding, the availability of specific reversal agents like idarucizumab for dabigatran enhances the safety profile, though reversal strategies remain more challenging for factor Xa inhibitors in the context of liver failure [6,181].

Importantly, the benefits of anticoagulation appear to extend beyond stroke prevention. Evidence indicates that appropriate anticoagulation in AF patients with compensated cirrhosis may be associated with a reduced risk of hepatic decompensation and improved survival [144]. This highlights that effective systemic anticoagulation might mitigate some of the prothrombotic drivers of portal hypertension and disease progression.

When interpreting the observational evidence summarized in this review, several methodological considerations merit acknowledgment. First, confounding by indication is inherent in non-randomized comparisons: patients prescribed DOACs versus warfarin may differ systematically in ways that influence outcomes (e.g., perceived bleeding risk, renal function, adherence patterns), potentially altering estimates of treatment effects. Second, immortal time bias can arise in retrospective cohort studies where anticoagulation status is treated as time-fixed rather than time-varying, potentially inflating apparent treatment benefits. Third, heterogeneity in cirrhosis severity classification across studies (ranging from ICD code-based definitions to verified Child–Pugh scores) limits comparability and may obscure differential treatment effects by disease stage. Finally, detection bias may occur if patients on warfarin (requiring regular INR monitoring) have more frequent healthcare contacts, leading to increased event detection compared to DOAC-treated patients. While these limitations do not negate the consistent signal favoring DOACs in compensated cirrhosis, they underscore the need for prospective studies with rigorous design and standardized severity classification.

Several critical gaps in knowledge persist. First, patients with significant hepatic impairment were excluded from the landmark RCTs establishing DOAC efficacy and safety. The current evidence is largely derived from retrospective cohort studies and meta-analyses thereof, which are susceptible to selection bias and confounding [98,176]. Second, there is an urgent need for validated, liver-specific bleeding risk scores that incorporate variables like platelet count, presence of varices, and albumin level, moving beyond the HAS-BLED score, which has limitations in this population [187]. Third, the role of anticoagulation in patients with advanced cirrhosis (Child–Pugh C) and AF remains profoundly uncertain and warrants dedicated study.

In conclusion, while DOACs have emerged as a promising therapeutic option for stroke prevention in AF patients with compensated liver cirrhosis, their use demands a nuanced, patient-centered approach. The Child–Pugh classification remains a cornerstone for guiding therapy. For now, in the absence of definitive RCT data, management must be individualized, weighing the specific risks and benefits for each patient, and should be orchestrated within a multidisciplinary framework. Future prospective, randomized trials with well-defined cirrhosis stages are essential to solidify evidence-based guidelines for this growing and complex patient population.

## 7. Conclusions

Acknowledging the important interplay between liver and cardiac diseases underscores the urgent need for comprehensive, randomized controlled trials that utilize standardized anticoagulation regimens and clearly define cirrhosis severity. Comprehensive studies are required to accurately assess the safety and efficacy of anticoagulant therapies in this complex patient population. Emerging evidence suggests that DOACs offer a favorable safety and efficacy profile compared to traditional anticoagulants like warfarin, particularly in patients with compensated (Child–Pugh A and some B) cirrhosis. In these populations, DOACs have been associated with lower rates of ischemic stroke and all-cause mortality, without a significant increase in major bleeding events. However, in patients with decompensated (Child–Pugh C) cirrhosis, DOACs remain contraindicated, and treatment decisions must be individualized based on liver function, bleeding risk, and thromboembolic risk.

To optimize anticoagulation management in these patients, there is a pressing need to develop bleeding and thromboembolic risk scores specifically tailored to patients with LC. In addition, the integration of biomarkers predictive of bleeding complications into clinical practice could help individualize treatment, particularly in patients with decompensated cirrhosis, where therapeutic decisions are especially challenging.

Current evidence highlights the need for clinicians to recognize the concurrent risks of both thromboembolic and bleeding complications in patients with atrial fibrillation (AF) and LC, in order to propose specific, evidence-based guidelines for managing these complex patients. Ultimately, future randomized controlled trials with well-defined cirrhosis stages and standardized anticoagulation protocols are essential to guide clinical decision-making. Until then, a multidisciplinary, individualized approach remains critical in managing patients with both AF and LC.

## Figures and Tables

**Table 1:** Metabolism pathways of oral anticoagulants.

Anticoagulant	Hepatic Metabolization	Renal Excretion
Warfarin	100%	0
Apixaban	75%	25%
Dabigatran	20%	80%
Rivaroxaban	65%	35%
Edoxaban	50%	50%

**Table 2:** Summary of meta-analyses comparing DOACs and warfarin in patients with atrial fibrillation and liver cirrhosis.

Reference	Authors and Year	# of Studies/ Participants	Study Design	Outcomes Reported	Effect Measures Used	Key Findings (DOACs vs. Warfarin/VKAs/LMWH)	Notes/ Key Limitations
[167]	Zhao et al. (2023)	18/41,447	Meta-analysis of observational and RCT data	All bleeding, major bleeding, ICH, GI bleeding, all-cause death	RR, 95% CI	Significant reduction in all bleeding, major bleeding, ICH, GI bleeding, and all-cause death. Benefits in mild–moderate cirrhosis.	Includes broad liver disease severity.
[168]	Menichelli et al. (2021)	12/43,532	Meta-analysis of cohort and RCT data	Major bleeding, ICH, any bleeding, GI bleeding, rDVT, death, IS/SE	HR, 95% CI	Major bleeding ↓61%, ICH ↓52%, rDVT ↓82%. No difference in death or IS/SE.	Focus on advanced disease; no GI bleeding difference.
[169]	Huang et al. (2021)	6/41,859	Meta-analysis of observational studies	Ischemic stroke, major bleeding, ICH, GI bleeding	HR, 95% CI	Ischemic stroke ↓, major bleeding ↓, ICH ↓. No GI bleeding difference. Dabigatran and apixaban safer.	Focus on AF; includes dose subgroups.
[170]	IbnE Ali Jaffari et al. (2024)	8/20,684	Meta-analysis of observational studies	All-cause death, ischemic stroke, major bleeding, GI bleeding, ICH	RR, 95% CI	All-cause death ↓, ischemic stroke ↓, major bleeding ↓, ICH ↓. GI bleeding non-significant.	High heterogeneity in some outcomes.
[171]	Lee et al. (2022)	3/4011	Meta-analysis of retrospective studies	Ischemic stroke, major bleeding, ICH	HR, 95% CI	Ischemic stroke ↓, major bleeding ↓, ICH ↓.	Small number of studies; all retrospective.
[172]	Zhou et al. (2025)	14/44,848	Meta-analysis of observational and RCT data	Major bleeding, ICH, GI bleeding, all-cause death, ischemic stroke/SE	RR, 95% CI	Major bleeding ↓, ICH ↓, GI bleeding ↓, death ↓. No difference in ischemic stroke/SE. Apixaban safer.	Large sample; includes recent studies up to 2024.
[173]	Sinha et al. (2024)	10/N/A	Meta-analysis of observational studies	Stroke/SE, all-cause death, major bleeding	RR, 95% CI	Stroke/SE ↓, major bleeding ↓. No mortality difference.	Focus on cirrhosis; does not report total n.
[174]	Hu et al. (2023)	7/7551	Meta-analysis of cohort studies	Ischemic stroke/SE, all-cause death, major bleeding, ICH, major GI bleeding	HR, 95% CI	Major bleeding ↓, ICH ↓, GI bleeding ↓. No difference in stroke/SE or death. Benefits in advanced cirrhosis.	Updated review; focuses on cirrhosis only.
[175]	Hoolwerf et al. (2018)	5/239	Systematic review (no meta-analysis)	VTE/SVT progression, major bleeding, all-cause death	Descriptive statistics	DOACs appear effective and safe; major bleeding 4–15% vs. 7–28% with VKAs/LMWH.	Small n; heterogeneous studies; no pooled analysis.
[176]	Chokesuwattanaskul et al. (2019)	7/19,798	Meta-analysis of cohort studies	Stroke, bleeding	HR, 95% CI	Anticoagulation reduces stroke without increasing bleeding. DOACs safer than warfarin.	Compares anticoagulation vs. none; indirect DOAC comparison.
[177]	Nisly et al. (2021)	7/683	Meta-analysis of observational studies	ISTH major bleeding, all bleeding, ICH, GI bleeding	OR, 95% CI	No significant difference in ISTH major bleeding. Similar safety profile.	Strict bleeding definition; small sample; mild-moderate only.

## Data Availability

No new data were created.
